# Effects of Ankle Joint Angles and Surrounding Muscles on Hip Joint Musculature

**DOI:** 10.3390/jfmk10020110

**Published:** 2025-03-27

**Authors:** Yuta Murata, Noriyuki Kida, Takumi Jiromaru, Michio Wachi, Kohei Yoshikawa, Shinichi Noguchi, Hitoshi Onishi

**Affiliations:** 1 Furu Clinic, 1098 Konancho Terasho, Koka 520-3301, Japan; yutapt1453@gmail.com; 2 Graduate School of Science and Technology, Kyoto Institute of Technology, Kyoto 606-8585, Japan; kohei.yoshikawa.601520@gmail.com; 3 Faculty of Arts and Sciences, Kyoto Institute of Technology, Kyoto 606-8585, Japan; 4 Department of Physical Therapy, School of Health Science, Bukkyo University, Kyoto 604-8418, Japanm-wachi@bukkyo-u.ac.jp (M.W.); 5 Kanazawa Orthopaedic and Sports Medicine Clinic, 881 Ono, Ritto 520-3016, Japan; 6 Department of Physical Therapy, School of Rehabilitation, Biwako Professional University of Rehabilitation, Higashiomi 527-0145, Japan; s-noguchi@pt-si.aino.ac.jp (S.N.); h-onishi@pt-si.aino.ac.jp (H.O.)

**Keywords:** hip abduction, gluteus medius, myofascial chains

## Abstract

**Background/Objectives**: Hip abductor weakness is a common issue in patients with lower back pain, knee osteoarthritis, and hip disorders, and compromises pelvic stability, gait control, and function. Side-lying hip abduction exercises are widely used as safe and effective interventions for patients unable to perform high-load or weight-bearing activities. However, the influence of ankle joint angles and distal muscle activity on the hip abductor muscles remains unclear. This study aimed to investigate the effects of ankle joint angles and activation states on unilateral right hip abductor strength and muscle activity. **Methods**: Fifteen healthy male adults (29.1 ± 5.4 years) participated. Surface electromyography (EMG) was used to measure the activity of the tensor fasciae latae (TFL), gluteus medius (G-med), gluteus maximus, tibialis anterior, and medial gas-trocnemius muscles. Hip abduction strength was evaluated in a side-lying position with the ankle positioned at three angles (neutral, dorsiflexion, and plantarflexion) and in three activation states (no activation, maximal dorsiflexion, and maximal plantarflexion). Two-factor (3 × 3) repeated measures ANOVA was used to analyze strength and EMG activity. **Results**: ANOVA revealed a significant interaction effect. The results of the simple main effects showed significantly higher hip abduction strength in dorsiflexion than in the neutral position and plantarflexion (*p* < 0.001). TFL and G-med EMG activities peaked during dorsiflexion, particularly under maximal dorsiflexion. **Conclusions**: These findings suggest that dorsiflexion enhances hip abductor strength and activity by increasing fascial tension (lateral line and superficial backline) and improving limb alignment. This approach may provide effective rehabilitation strategies. This is a load-adjustable training recovery approach that should be confirmed with future intervention studies.

## 1. Introduction

Muscle weakness in the hip abductors is a significant factor contributing to reduced pelvic stability and impaired control of the movement of the center of gravity during walking. This condition is often associated with musculoskeletal disorders such as low back pain, knee osteoarthritis, and hip joint diseases, thereby increasing the risk of further functional impairments [1,2,3]. Studies on the postoperative outcomes of hip osteoarthritis have shown that hip abductor weakness directly correlates with altered gait patterns and functional limitations [4], while atrophy of the gluteal muscles is linked to the progression and severity of the disease [5]. These findings strongly suggest that dysfunction of the hip musculature affects not only local joint stability but also overall motor function.

The fascia, a connective tissue that links muscles, tendons, bones, and nerves, plays a critical role in transmitting tension throughout the body [6]. Myofascial chains, such as the lateral line and superficial backline, mediate tension transfer from the ankle to the hip, thereby enhancing dynamic stability [7]. The fascia also contains proprioceptors that relay sensory information to the central nervous system, thereby coordinating motor control across the body [8]. Understanding the interplay between the myofascial chains and neural facilitation is expected to lead to innovative rehabilitation approaches aimed at improving motor control and functional recovery.

Myofascial chains refer to continuous connective tissue linkages between different body regions and play vital roles in motor function, force transmission, postural control, and body stability. The fascia envelops the muscles, tendons, ligaments, and bones, forming a network that facilitates force transmission, influencing intersegmental dynamics, and transmits sensory inputs. Myofascial chains, including the lateral line and superficial backline, transmit forces from the ankle to the hip and trunk, contributing not only to dynamic stability and movement efficiency but also to the mechanisms underlying pain and movement dysfunction. Proprioceptors within the fascia transmit sensory information to the central nervous system, highlighting the potential for improved movement efficiency and rehabilitation outcomes through the exploration of myofascial chains and neural facilitation. These properties of the myofascial chains serve as a theoretical foundation for holistic interventions in rehabilitation and training. Recently, attention has been paid to evaluating these factors under non-weight-bearing conditions, as they offer a unique approach to assessing biomechanical and neurological factors in isolation [6].

Proprioceptive neuromuscular facilitation (PNF) therapies strive to stimulate muscle proprioceptors, such as muscle spindles and Golgi tendon organs, to enhance neuromuscular activation. This technique utilizes sensory input from the periphery to activate spinal reflexes and increase cortical excitability, thereby amplifying muscle activity [8,9]. A notable example is the “overflow phenomenon”, where the contraction of one muscle group extends to adjacent or distal muscle groups, enhancing overall muscular activity. For instance, tibialis anterior contraction during ankle dorsiflexion influences the quadriceps and hip abductor muscles [8,10]. Understanding the interaction between the PNF and myofascial chains is crucial for elucidating how the movements of one joint affect adjacent or distant joints, thus providing insights into the influence of ankle dorsiflexion on hip abductor activity.

Factors such as ankle stability and angle are essential components in hip abductor training. Although the influence of ankle movements on the knee and hip has been studied primarily under weight-bearing conditions [11,12], external loads and gravitational effects during such conditions complicate the interpretation of fascial and neural mechanisms [13,14]. In contrast, non-weight-bearing conditions eliminate these external influences, enabling a more focused evaluation of the myofascial chains and neural facilitation. However, research in this area is limited.

Previous studies have often focused on intersegmental dynamics under weight-bearing conditions, where external forces and gravity influence the ankle and knee joints, making it challenging to isolate and evaluate intrinsic mechanisms, such as myofascial chains and neural facilitation. Non-weight-bearing conditions allow a clearer understanding of the involvement of the fascia and neural functions.

This study aimed to investigate the effects of distal ankle angles and muscle activity on hip musculature under non-weight-bearing conditions, thereby providing novel insights into the roles of myofascial chains and neural functions. Although traditional hip abductor-strengthening exercises often involve high-load resistance training in standing or weight-bearing positions, such exercises may not be safe or feasible for elderly individuals or patients with musculoskeletal disorders [15]. In contrast, side-lying hip abduction exercises offer a safe and low-load alternative to high-resistance weight-bearing exercises [16].

Additionally, this study explored how ankle angle and muscle activity influence hip abductor activity through myofascial chains with the goal of establishing a foundation for safe and effective rehabilitation strategies for elderly individuals and patients with musculoskeletal disorders. By deepening the understanding of kinetic chains under non-weight-bearing conditions, this study aims to propose widely applicable training methods for clinical practice.

## 2. Materials and Methods

### 2.1. Participants

Fifteen healthy adult males (mean age: 29.1 ± 5.4 years; height: 172.3 ± 5.7 cm; mass: 66.9 ± 9.4 kg) participated in this study. Participants with a history of pain, significant postural abnormalities, or major neurological or respiratory conditions were excluded. Informed consent was obtained from all the participants. This study was approved by the ethics committee of the Kanazawa Orthopedic Surgery Medical Clinic (Kanazawa-OSMC-2024-006) and was conducted in accordance with the Declaration of Helsinki. The study design, participants, and inclusion/exclusion criteria were reported in accordance with the STROBE guidelines.

### 2.2. Electromyography (EMG) Recording

Excess hair was shaved, and careful skin preparation was performed to reduce skin impedance to below 5 kΩ. After drying the skin, pairs of Ag/AgCl surface electrodes (ELRSA20, SMP-300; METS Co., Ltd., Tokyo, Japan, diameter: 10 mm) were placed with an inter-electrode distance of 10 mm at the following locations. The electrodes were placed at five sites: for the tensor fasciae latae (TFL), parallel to the muscle fibers and approximately 2 cm below the anterior superior iliac spine with the leg extended; for the posterior fibers of the gluteus medius (G-med), at one-third of the proximal distance between the iliac crest and the greater trochanter, anterior to the gluteus maximus (G-max), aligned with the muscle fibers; for the lower fibers of G-max, at the midpoint between the greater trochanter and the sacrum (S3) at the level of the trochanter, parallel to the muscle fibers [17]; for the tibialis anterior, four finger-widths distal to the tibial tuberosity and one finger-width lateral to the tibial crest [18]; and for the medial head of the gastrocnemius, five finger-widths distal to the popliteal crease on the medial muscle belly [19]. The EMG signals were differentially amplified, sampled at 1000 Hz with 12-bit resolution, and A/D converted for recording and storage on a computer using VitalRecorder2 ver. 3.8.4. 1403 software (KISSEI COMTEC Co., Ltd., Nagano, Japan).

### 2.3. Experimental Tasks

**Figure 1:**
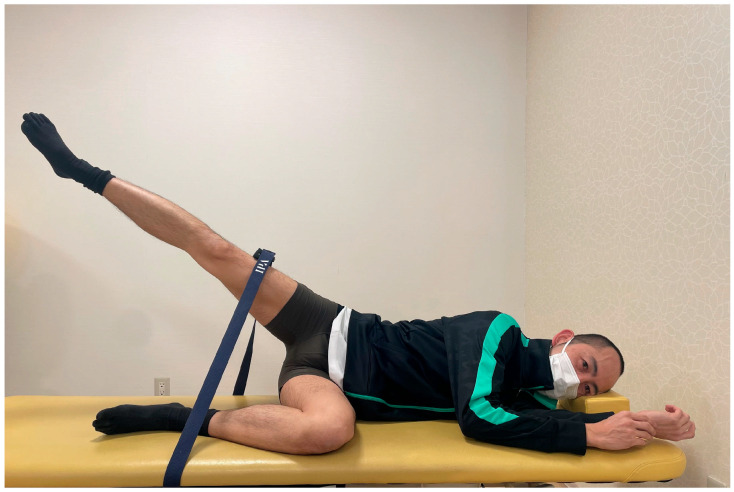
Experimental design of the hip abduction task.

All participants performed maximal hip abduction of the tested lower limb in the side-lying position. Hip abductor strength was measured using a handheld dynamometer (mobie2, SAKAImed Co., Ltd., Tokyo, Japan) placed between the belt and the lateral thigh. Hip abduction movement was performed as a resisted movement against the belt in the maximum hip abduction position. The dynamometer was calibrated before each measurement to ensure accuracy. The validity and reliability of handheld dynamometry for measuring lower-extremity muscle strength are reported in previous research [20]. While isokinetic dynamometry is considered the gold standard for muscle strength assessment, handheld dynamometers provide a practical alternative for isometric strength testing. In this study, the peak force value was manually recorded. This dynamometer “fixed” the maximum force value. To provide a clearer understanding of the handheld dynamometer measurement process, we will include a photograph of the measurement (Figure 1). The distal lateral thigh was secured to the bed using a belt to ensure maximal isometric contraction in the hip abduction position. The non-tested lower limb was flexed at the hip and knee to approximately 90°. The trunk was positioned neutrally, and the participants were instructed not to press against the bed with their upper limbs. The experimental conditions included three ankle joint angles (neutral, dorsiflexion, and plantarflexion) combined with three force conditions (no force, maximal dorsiflexion force, and maximal plantarflexion force), resulting in nine conditions. The tasks were performed in a randomized order, and hip abductor strength measurements were performed in a randomized order. Each task was performed twice for 5 s, with adequate rest between trials, and the average of the two trials was used. Participants performed maximal resistance movement against the belt under the nine conditions of ankle angle and force exertion. The hip abduction angle was the same as that used for the maximum voluntary isometric contraction (MVIC) of the hip abductors. Additionally, the maximum muscle activity of the TFL, gluteus medius, and gluteus maximus was measured in the position used for manual muscle testing by Daniels et al. The participants also performed maximal voluntary isometric contraction (MVIC) of each muscle for 5 s against belt resistance. MVIC trials were repeated twice with sufficient rest, and the higher value was used. This study ensured consistency by using standardized equipment, positioning, and measurement protocols.

### 2.4. Data Analysis

The EMG data were processed using analysis software (KineAnalyzer VitalRecorder2 ver. 3.8.4. 1403, KISSEI COMTEC Co., Ltd., Nagano, Japan). Raw signals were bandpass-filtered between 10 and 500 Hz and fully rectified. The root mean square was calculated using a 300 ms window for the hip abduction tasks and MVIC trials under each of the nine conditions. The root mean square values were normalized based on the MVC obtained during manual muscle testing. The data analysis process followed predefined protocols to ensure reproducibility and consistency.

### 2.5. Statistical Analysis

Hip abductor strength and hip muscle activity across the measurement positions were compared using repeated measures analysis of variance (ANOVA), examining the effects of ankle joint angles (three levels: dorsiflexion, neutral, and plantarflexion) and muscle force exertion conditions (three levels: dorsiflexion effort, no effort, and plantarflexion effort) on four dependent variables, hip abduction strength, TFL activity, G-med activity, and G-max activity, which served as the dependent measures. The simple main effects for each condition were tested. Multiple comparisons of the means were conducted using estimated marginal means, with Bonferroni correction applied to adjust the significance level. Statistical significance was set at *p* < 0.05. All analyses were performed using SPSS ver. 29.0.2 (IBM Corp., Armonk, NY, USA). To prevent the inflation of Type I errors due to multiple ANOVAs, we applied Holm’s stepwise correction method. Additionally, multiple comparisons were performed using the Tukey HSD test to further validate the pairwise differences across the conditions. This approach ensured that both family-wise error rates and individual pairwise comparisons were adequately controlled, providing robust statistical reliability for our findings.

## 3. Results

**Figure 2:**
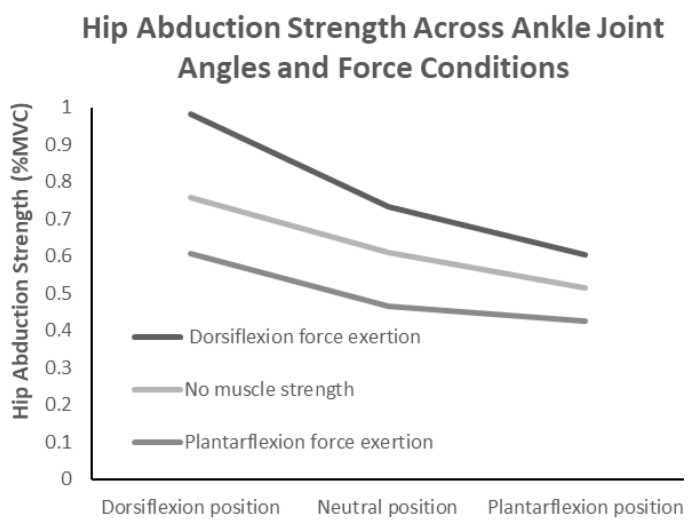
Interaction effects of ankle joint position and muscle force exertion on hip abductor strength.

Table 1 shows that the hip abductor strength values according to the ankle joint angles and muscle force exertion conditions were ordinal (Figure 2). A two-way repeated measures ANOVA, with ankle joint angle (three levels) and ankle muscle force exertion condition (three levels) as within-subject factors, revealed a significant interaction effect (F(4,56) = 7.978, *p* < 0.001, η^2^ = 0.363). An inspection of the interaction plots indicated that the interaction was ordinal. A subsequent simple main effects analysis showed that for all muscle force exertion conditions, hip abductor strength was significantly higher in the neutral position than in plantarflexion and significantly higher in dorsiflexion than in the neutral position. Additionally, for all ankle joint angle conditions, the strength values were significantly higher without force exertion than with plantarflexion force exertion and significantly higher during dorsiflexion force exertion than without force exertion. Furthermore, multiple comparisons using the Holm method identified nine significant pairs, and the Tukey HSD test confirmed that all eight pairs remained significant. These results suggest that both ankle joint angle and muscle force exertion conditions play crucial roles in modulating hip abductor strength. In particular, dorsiflexion force exertion consistently led to the highest strength values, while plantarflexion force exertion resulted in the lowest. Table 1 summarizes the hip abduction strength and hip muscle activity values across the conditions.

**Figure 3:**
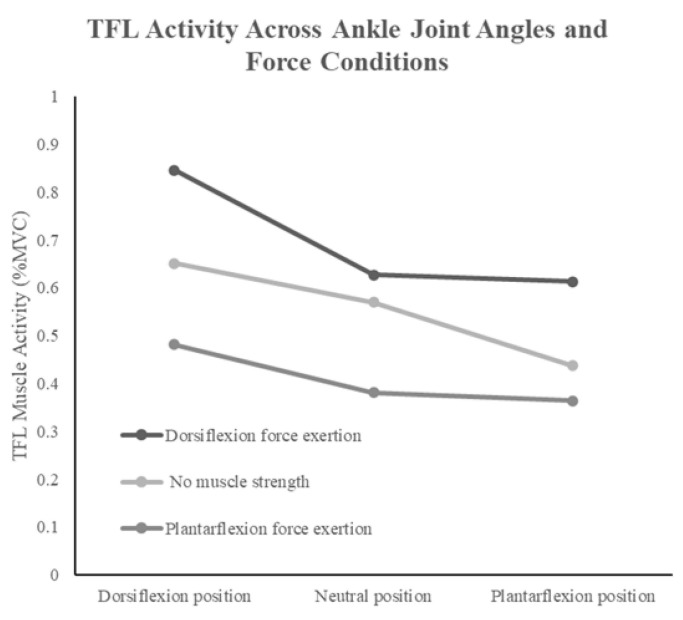
Interaction effects of ankle joint position and muscle force exertion on tensor fasciae latae activity.

For TFL, two-way repeated measures ANOVA revealed a significant interaction effect (F(4,56) = 3.563, *p* < 0.012, η^2^ = 0.203). The interaction was also ordinal, and simple main effects analysis demonstrated that TFL activity was significantly higher in the neutral position than in plantarflexion and significantly higher in dorsiflexion than in the neutral position for all muscle force exertion conditions. Moreover, TFL activity was significantly higher without force exertion than with plantarflexion force exertion and significantly higher during dorsiflexion force exertion than without force exertion. Additionally, multiple comparisons using the Holm method identified five significant pairs, all of which remained significant in the Tukey HSD test. These results indicate that TFL activity was highest during dorsiflexion force exertion and lowest during plantarflexion force exertion. (Figure 3). Moreover, across all ankle joint angle conditions, TFL activity was significantly higher particularly during dorsiflexion force exertion.

**Figure 4:**
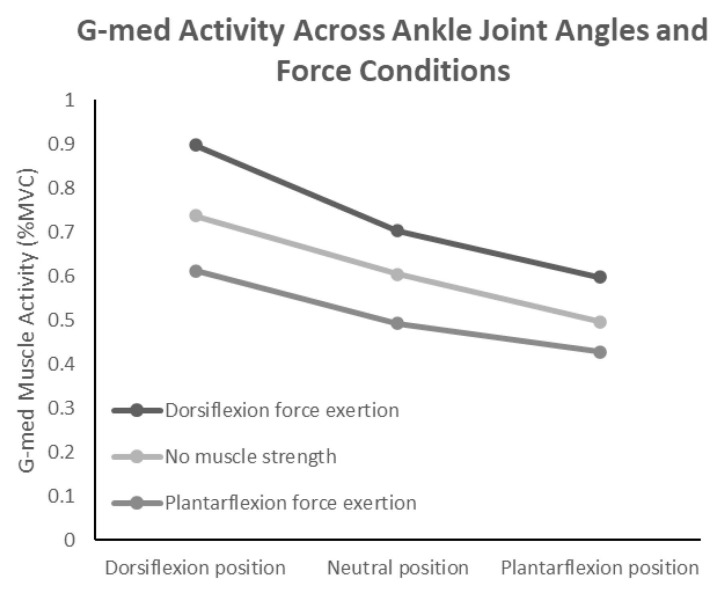
Interaction effects of ankle joint position and muscle force exertion on gluteus medius activity.

For G-med, the two-way repeated measures ANOVA also showed a significant interaction effect (F(4,56) = 3.247, *p* < 0.018, η^2^ = 0.188). The interaction was also ordinal, and simple main effects analysis revealed that under conditions of no force exertion and dorsiflexion force exertion, G-med activity was significantly higher in the neutral position than in plantarflexion and significantly higher in dorsiflexion than in the neutral position. Under the plantarflexion force exertion condition, G-med activity was significantly higher in dorsiflexion than in both the neutral and plantarflexion positions. Furthermore, multiple comparisons using the Holm method identified eight significant pairs, all of which remained significant in the Tukey HSD test. These results indicate that G-med activity was highest during dorsiflexion force exertion and lowest during plantarflexion force exertion. (Figure 4). Additionally, across all ankle joint angle conditions, G-med activity was significantly higher particularly during dorsiflexion force exertion. Moreover, a separate analysis extracting non-duplicated comparisons revealed eight distinct pairs that demonstrated significant differences (*p* < 0.05).

For G-max, no significant interaction effect was observed in the two-way repeated measures ANOVA (F(4,56) = 1.857, *p* = 0.131, η^2^ = 0.117). However, simple main effects analysis indicated that under conditions of no force exertion and dorsiflexion force exertion, G-max activity was significantly higher in the neutral position than in plantarflexion and significantly higher in dorsiflexion than in the neutral position. Furthermore, in terms of ankle joint angle conditions, activity was significantly higher without force exertion than with plantarflexion force exertion in both dorsiflexion and plantarflexion and significantly higher during dorsiflexion force exertion than without force exertion. In the no-force condition, G-max activity was significantly higher during dorsiflexion force exertion than without force exertion. Additionally, multiple comparisons using the Holm method identified five significant pairs, all of which remained significant in the Tukey HSD test. These results suggest that G-max activity increases particularly during dorsiflexion force exertion across multiple ankle joint angles, while consistently remaining lowest during plantarflexion force exertion. Furthermore, among the comparisons involving the same ankle joint (dorsiflexion), five pairs showed significant differences (*p* < 0.05).

## 4. Discussion

### 4.1. Relationship Between Ankle Joint Angle and Hip Musculature

The results of this study demonstrated that ankle dorsiflexion and plantarflexion significantly influenced hip muscle activity. Hip abductor strength was significantly higher in dorsiflexion than in the neutral and plantarflexion positions. Similarly, the muscle activities of TFL, G-med, and G-max were significantly greater in dorsiflexion than in the neutral or plantarflexion positions. All muscles analyzed via EMG in this study were involved in hip abduction, and their activity was significantly enhanced during dorsiflexion compared to the other positions. These results suggest that ankle joint angles are associated with the activity level of the hip abductors during hip abduction. Furthermore, the same results were obtained under the no-force exertion condition, which excluded the effects of muscle output. Under these conditions, it was possible to evaluate the effects purely based on the joint angles and neuromuscular mechanisms, clearly demonstrating that ankle joint angles affect hip abductor activity.

The novelty of this study lies in its examination of the effects of ankle dorsiflexion on hip musculature under non-weight-bearing conditions, thereby elucidating the roles of the myofascial chains and neural functions. While previous studies have primarily focused on kinetic chains under weight-bearing conditions, it is challenging to accurately evaluate internal mechanisms such as myofascial chains and neural facilitation, owing to the influence of external loads and gravity on the ankle and knee joints. In contrast, this study eliminated these external influences by employing non-weight-bearing conditions, thus clarifying the mechanisms involved in the pure involvement of fascial and neural functions. Additionally, previous research has shown similar distal-to-proximal influences in the upper extremity joints, suggesting that mechanical effects from the distal to the proximal regions play a critical role in motor function [8]. This study provides evidence that ankle dorsiflexion under non-weight-bearing conditions facilitates tension transfer from the distal (ankle joint) to the proximal (hip joint) regions via myofascial chains, enhancing the activity of the hip abductor muscles.

These findings align with the principles of myofascial connectivity, where fascial tension generated at the distal segments propagates along interconnected kinetic pathways, influencing proximal muscle activation patterns. The enhanced activation of the hip abductors observed in dorsiflexion may also be attributed to improved neuromuscular efficiency in coordinating distal and proximal muscle groups, supporting the hypothesis that dorsiflexion promotes optimal force distribution throughout the lower limb kinetic chain. Furthermore, the observed increase in hip abductor strength in dorsiflexion reinforces the idea that targeted dorsiflexion interventions could play a crucial role in rehabilitation and strength training protocols.

During dorsiflexion, the lateral and superficial back lines are stretched, generating fascial tension that enhances the activities of TFL, G-med, and G-max via the iliotibial band. This tension transfer through the myofascial chains contributes not only to localized muscle activity but also to efficient kinetic chains from the distal to proximal regions, increasing the activity of the hip musculature. In contrast, fascial tension diminishes during plantarflexion, leading to insufficient support through fascial mechanisms, potentially limiting muscle output. The observed significant reduction in muscle activity during plantarflexion compared to dorsiflexion supports the hypothesis that the lack of fascial stretch impairs effective tension transfer. These findings strongly support the role of dorsiflexion in activating the myofascial chains and improving muscle activity.

The increased muscle activity during dorsiflexion can be attributed to the tension transfer of myofascial chains, specifically the lateral and superficial back lines, which facilitate interactions among the ankle, knee, and hip joints, resulting in enhanced hip abductor strength [21,22]. Specifically, increased tension in these myofascial chains may have been efficiently transmitted via the iliotibial band, thereby enhancing the activity of TFL and G-med [23,24].

Neural elongation is another factor that influences muscle activity. Under the conditions in this study, maximal dorsiflexion likely placed tension on the entire sciatic nerve, including the inferior gluteal nerve, which may have affected the output of G-max. This phenomenon aligns with previous research indicating that mechanical stress on axons and myelin during neural elongation can alter the conduction efficiency of action potentials [25]. While moderate neural elongation (10–15%) may not significantly affect conduction, excessive elongation can impair nerve conduction, leading to decreased muscle output due to elevated intraneural pressure and reduced blood flow [25,26]. This study uniquely demonstrated that dorsiflexion increases tension on the sciatic nerve, specifically suppressing G-max output through this mechanism.

In contrast, the superior gluteal nerve has a relatively short pathway from the sciatic nerve to G-Med and TFL, making it less affected by dorsiflexion-induced neural elongation. G-med functions as the primary hip abductor and can maintain a stable force output within the length–tension curve, even during maximal hip abduction [25]. Therefore, G-med and TFL likely maintain efficient activity during dorsiflexion, contributing to hip abductor strength. In contrast, the lower G-max activity compared with that of the other muscles may have resulted from dorsiflexion-induced neural elongation, specifically affecting the inferior gluteal nerve.

By adopting a side-lying position, this study created an environment that minimized the effects of gravity while maximizing pelvic and hip muscle activities, allowing for a detailed examination of the relationship between neural elongation and muscle activity. This approach provided a foundation to elucidate the effects of dorsiflexion and hip abduction on neural conduction and muscle output regulation.

This mutual facilitative effect can be explained through neuromuscular coordination mechanisms, where dorsiflexion force exertion recruits proprioceptive feedback systems that enhance lower limb stability. The increased activation of the tibialis anterior and associated dorsiflexors during dorsiflexion force exertion may induce the cross-facilitation of hip musculature via neural pathways, particularly through the interplay of spinal reflex arcs and central nervous system regulation. This interaction between distal and proximal muscle activation is fundamental in motor control and rehabilitation, as it highlights the importance of integrated neuromuscular strategies rather than isolated muscle strengthening. Additionally, dorsiflexion-driven force exertion may influence intermuscular coordination by modifying lower limb biomechanics, promoting alignment that is conducive to efficient force application during functional movements.

The results of this study demonstrate that ankle dorsiflexion increases tension on the sciatic nerve, resulting in differential effects on lower limb muscle output. These findings highlight the mechanism by which muscle activity is selectively modulated based on neural innervation specificity and offer valuable insights into neuromuscular function under non-weight-bearing conditions.

### 4.2. Relationship Between Ankle Joint Muscle Force and Hip Musculature

This study investigated the effects of dorsiflexion and plantarflexion force exertion of the ankle joint on hip abductor strength and surrounding muscle activity in non-weight-bearing side-lying conditions. The results revealed that hip abductor strength was significantly higher during dorsiflexion force exertion than during no-force exertion or plantarflexion force exertion across all ankle joint angles.

These findings suggest that ankle dorsiflexion is a key factor in enhancing hip muscle activity. Previous studies have shown that increased tension in distal muscle groups, as part of a PNF pattern, promotes central and proximal muscle activation through reflexive neural facilitation mechanisms mediated by muscle spindles and Golgi tendon organs [8,27,28]. However, in this study, despite the increased distal muscle tension during plantarflexion force exertion, the activity of the hip abductor muscles did not reach optimal levels. This discrepancy may be attributed to the specific neural roles and functional characteristics of the plantarflexor muscles.

During plantarflexion force exertion, the primary muscles involved, the gastrocnemius and soleus, are responsible for the localized stabilization of the ankle and knee joints. This limits the directionality of neural facilitation and may prevent the effective activation of the kinetic chain from distal to proximal regions. Additionally, the tension inhibition mechanisms mediated by the Golgi tendon organs during the contraction of the gastrocnemius and soleus may restrict the transmission of neural facilitation to the knee and hip joints [29].

The interaction between ankle joint force exertion and joint positioning demonstrates a synergistic effect on hip abductor activation, with dorsiflexion force exertion in a dorsiflexed position enhancing neuromuscular engagement at the hip. This combination of muscle force exertion and joint configuration optimizes neuromuscular coordination, highlighting the importance of integrated effects rather than relying solely on isolated muscle recruitment. The concurrent activation of distal and proximal muscle groups suggests that dorsiflexion force exertion acts as a functional stimulus, potentially strengthening motor control strategies that integrate segmental force transmission.

Dorsiflexion force exertion also facilitates crucial neuromuscular coordination in hip muscle activation and may support an optimal length–tension relationship in the hip abductors. This, in turn, enhances the stability and efficiency of force transmission from distal to proximal segments, with the consistent activation of the hip abductors under dorsiflexion force exertion indicating that this mechanism is part of a broader neuromuscular regulation strategy.

In contrast, dorsiflexion force activates the tibialis anterior, extensor hallucis longus, and extensor digitorum longus, triggering stretch reflexes via the Ia afferent nerve fibers from the muscle spindles. These reflexes enhance agonist muscle contraction while reducing the activity of the antagonist gastrocnemius and soleus muscles through reciprocal inhibition, enabling the efficient transmission of neural facilitation from distal to proximal regions. Furthermore, reflexive excitatory connections between the tibialis anterior and quadriceps muscles may induce an overflow phenomenon, further enhancing hip abductor activity.

Under non-weight-bearing conditions, plantarflexion may destabilize the talocrural joint, negatively affecting postural control at the knee and hip joints. This instability can result in excessive knee extension or inappropriate hip rotation, impairing the optimal function of the quadriceps, G-med, and G-max. Conversely, dorsiflexion stabilizes the talocrural joint by firmly positioning the talus between the tibia and fibula, promoting natural knee extension and improving the coordination of knee and hip joint movements. The additional contraction of the quadriceps stabilizes the knee joint, enabling efficient force transmission throughout the lower limbs. This structure reduces excessive joint movement during hip abduction, thereby enhancing the efficiency of hip abductor activity.

Moreover, maintaining a knee extension increases the external moment arm during hip abduction, which increases the internal moment arm of G-med. This biomechanical alignment optimizes the activity of G-med, further supporting the role of ankle dorsiflexion in improving functional connectivity and the muscle activity efficiency of the lower limb [16,30].

The selection of the side-lying position in this study also contributed to these results. In this position, G-med is nearly perpendicular to the gravitational axis, whereas TFL assumes a slightly oblique angle. This alignment allows G-med to act as the primary agonist during hip abduction, while TFL is also well positioned to contribute. The increased activity of TFL during dorsiflexion observed in this study contrasts with previous findings that suggested decreased activity in knee-flexed positions [17]. This discrepancy may be due to dorsiflexion-inducing knee extension, which increases the mechanical loading on TFL, thereby enhancing its activity in opposition to gravity. Additionally, G-med, which is nearly perpendicular to the side-lying position, operates efficiently with maximized moment arms, further enhancing its activity during hip abduction [30].

This suggests the potential application of these findings in clinical rehabilitation for conditions such as hip instability, neuromuscular impairments, and post-surgical motor function recovery. In these situations, the coordination between distal and proximal muscle functions may play a crucial role in mobility recovery and improving movement efficiency. Future research should investigate the long-term effects of interventions combining ankle joint angles and ankle force exertion conditions on functional outcomes, particularly in populations with impaired motor control or dysfunctional movement chains. These findings demonstrate a novel mechanism by which ankle dorsiflexion facilitates hip abductor activity via neural facilitation and distal-to-proximal neuromuscular connectivity. Specifically, the observation that dorsiflexion enhances hip muscle activity complements the existing research on kinetic chains under weight-bearing conditions and provides critical insights into rehabilitation and training strategies under non-weight-bearing conditions. This study underscores the potential of dorsiflexion-focused therapeutic exercises to improve lower-limb muscle function and kinetic chain efficiency, expanding the applicability of these approaches in rehabilitation and training programs.

### 4.3. Clinical Implications

The results of this study suggest that training incorporating ankle dorsiflexion and dorsiflexion force exertion can effectively enhance the activity of the hip abductor muscles and may serve as a novel intervention method for patients with hip joint disorders or osteoarthritis. Furthermore, by adjusting ankle joint angles and force exertion conditions, it is possible to appropriately modify the load, making this approach suitable for effective training and rehabilitation. Additionally, this method can be adapted for patients with weight-bearing restrictions, allowing for safe and progressive muscle strengthening. In dorsiflexion, the stability of the talocrural joint is improved, and neural facilitation is efficiently transmitted to the knee and hip muscles, maximizing the activity of the hip abductor muscles. This may indicate its potential to strengthen muscles and reduce joint stress, although further studies are required to confirm clinical effectiveness. Additionally, combining dorsiflexion force exertion further amplified neural facilitation, significantly improving lower limb stability and muscle activity efficiency. However, this study is based on a small sample of healthy males, and the results represent the neuromuscular phenomena observed in this limited group. Therefore, these findings support treatment hypotheses that need to be tested in future intervention studies, with further validation required.

Training under non-weight-bearing conditions eliminates the external loads and gravitational effects inherent in weight-bearing conditions, allowing for effective interventions that utilize the pure involvement of the myofascial chains and neural facilitation. This study demonstrated that ankle dorsiflexion under non-weight-bearing conditions is a safe method for eliciting sufficient muscle activity while avoiding excessive joint stress. This approach offers a practical method for enhancing muscle strength and reconstructing kinetic chains during the early stages of rehabilitation or in situations where weight bearing is restricted. Clinically, training that incorporates dorsiflexion and dorsiflexion force exertion is particularly useful in early postoperative rehabilitation, enabling the safe recovery of muscle function through gradually adjusted loads [31,32]. Moreover, side-lying hip abduction exercises minimize weight bearing, making them a safe and adaptable training method for elderly individuals or patients with concerns about muscle weakness. The combination of dorsiflexion force exertion and traditional dorsiflexion training enhances its neural facilitation effects, potentially improving the efficiency of rehabilitation.

However, placing the ankle in plantarflexion results in an unstable talocrural joint, leading to excessive activity of the ankle and knee-stabilizing muscles, which decreases overall lower limb joint stability. This instability was associated with significantly lower hip abductor strength and muscle activity compared to dorsiflexion and dorsiflexion force exertion, confirming reduced movement efficiency. These findings clearly demonstrate the influence of ankle joint angle and force exertion conditions on hip abductor muscle activity, reinforcing the role of dorsiflexion and dorsiflexion force exertion in enhancing lower limb stability and muscle activity efficiency [33].

Based on these results, training centered on ankle dorsiflexion and dorsiflexion force exertion proposes a novel mechanism of kinetic chain facilitation that promotes the efficient activity of the hip abductor muscles under non-weight-bearing conditions. These findings serve as new principles in rehabilitation and training design, offering clinical applications for improving joint stability and muscle activity efficiency in the lower limbs. Furthermore, this study highlights the utility of exercise therapy incorporating ankle dorsiflexion and dorsiflexion force exertion as an effective method for improving lower limb function and optimizing kinetic chain efficiency. This approach demonstrates flexibility, making it applicable even to patients with weight-bearing restrictions. However, it is important to note the limitation of the small sample of male, able-bodied participants in this study, who may not be representative of other populations, such as those differing in gender, age, or injury status.

### 4.4. Limitations

This study has several limitations. One limitation of this study is that it only examined one leg, without considering limb dominance. Limb dominance may influence muscle activation patterns, potentially affecting the results. To strengthen the findings, future studies should compare both legs and evaluate differences related to dominance. Including bilateral comparisons would provide a more comprehensive understanding of the effects observed in this study. Although the results demonstrated that ankle dorsiflexion under non-weight-bearing conditions promoted hip muscle activity through myofascial chains and neural facilitation, the consistency of these effects under different movement conditions or with varying loads remains unexamined. Understanding how the facilitation of muscle activity in dorsiflexion changes during dynamic movements or resistance is crucial for clinical applications. Furthermore, this study focused on short-term changes in muscle activity, and long-term follow-up studies are required to evaluate the extent to which continuous dorsiflexion training contributes to muscle strength improvement and functional recovery.

Finally, the specific mechanisms by which dorsiflexion enhances hip muscle activity via myofascial chains and neural facilitation cannot be fully explained based solely on the data from this study. Additionally, how these effects vary with exercise load, frequency, and intensity has not yet been thoroughly investigated. Further studies are required to clarify the detailed mechanisms.

Addressing these limitations using a multifaceted research approach is expected to enhance the effectiveness and applicability of dorsiflexion-based training. This will enable the design of safe and effective novel interventions for patients with muscle weakness and joint instability. Ultimately, this study could establish a foundation for new guidelines for training and rehabilitation, thereby offering significant improvements in clinical practice.

## 5. Conclusions

The results of this study revealed that combining dorsiflexion force exertion with ankle dorsiflexion during side-lying hip abduction exercises enhanced the activities of G-med, G-max, and TFL, contributing to increased hip abductor strength under controlled experimental conditions. Notably, G-med exhibited significantly greater activity than the other muscles, suggesting that dorsiflexion may be a promising approach for influencing hip muscle activation. While these findings provide insights into the neuromuscular mechanisms involved, they do not directly establish the effectiveness of dorsiflexion as a training or rehabilitation intervention. Future studies should investigate whether these neuromuscular responses translate into functional improvements in clinical or athletic populations.

## Figures and Tables

**Table 1:** Results of three factorial (3 × 3) repeated measures ANOVAs for hip abduction strength (%MIVC) and muscle activity (%MVC).

	Dorsiflexion Position			Neutral Position			Plantarflexion Position		
	DFX	NMS	PFX	DFX	NMS	PFX	DFX	NMS	PFX
	M ± SD	M ± SD	M ± SD	M ± SD	M ± SD	M ± SD	M ± SD	M ± SD	M ± SD
Hip abductor strength (%)	1.0 ± 0.0 *	0.7 ± 0.1 *	0.6 ± 0.1 *	0.8 ± 0.1 *	0.6 ± 0.1 *	0.5 ± 0.1 *	0.6 ± 0.1 *	0.5 ± 0.1 *	0.4 ± 0.1
Tensor fasciae latae (%)	0.8 ± 0.1 *	0.7 ± 0.1 *	0.5 ± 0.1	0.6 ± 0.2 *	0.6 ± 0.2 *	0.4 ± 0.1	0.6 ± 0.1 *	0.4 ± 0.2	0.4 ± 0.2
Gluteus medius (%)	0.9 ± 0.1 *	0.7 ± 0.1 *	0.6 ± 0.1 *	0.7 ± 0.1 *	0.6 ± 0.1 *	0.5 ± 0.1 *	0.6 ± 0.2 *	0.5 ± 0.2 *	0.4 ± 0.1
Gluteus maximus (%)	0.6 ± 0.2 *	0.4 ± 0.1 *	0.3 ± 0.2	0.4 ± 0.2 *	0.3 ± 0.1 *	0.3 ± 0.3	0.4 ± 0.2 *	0.3 ± 0.2	0.3 ± 0.2
**Main Effect**						**Interaction Effect**			
**Range**			**Muscle Strength**			**Range × Muscle Strength**			
**F**	***p***	**η** **^2^**	**F**	***p***	**η** **^2^**	**F**	***p***		**η** **^2^**
174.25	<0.001 *	0.926	196.08	<0.001 *	0.933	8.37	<0.001 *		0.374
29.93	<0.001 *	0.681	86.32	<0.001 *	0.86	3.56	0.012 *		0.203
57.61	<0.001 *	0.805	121.22	<0.001 *	0.896	3.25	0.018 *		0.188
10.99	<0.001 *	0.44	27.33	<0.001 *	0.661	1.86	0.131		0.117

The conditions in the dorsiflexion and neutral positions (with dorsiflexion force exertion and without force exertion) were significantly higher than those in plantarflexion and plantarflexion force exertion. * *p* < 0.05. DFX, dorsiflexion force exertion; NMS, no muscle strength; PFX, plantarflexion force exertion.

## Data Availability

The original contributions presented in this study are included in the article; further inquiries can be directed to the corresponding author.
